# Diagnostic potential of GLP recombinant antigens in whole blood assays for *Leishmania infantum* infection

**DOI:** 10.1186/s13071-025-07108-z

**Published:** 2025-11-21

**Authors:** Ana Victoria Ibarra-Meneses, Laura Fernández, Juan Víctor San Martín, Jesús García-Martínez, Laura Botana, Carmen Sánchez, Jose Carlos Solana, Lorena Bernardo, Steven G. Reed, Rhea N. Coler, Javier Moreno, Eugenia Carrillo

**Affiliations:** 1 Department Of Pathology and Microbiology, Faculty of Veterinary Medicine, Université de Montréal, Saint-Hyacinthe, QC Canada; 2 WHO Collaborating Centre for Leishmaniasis, National Centre for Microbiology, Instituto de Salud Carlos III, Majadahonda, Madrid Spain; 3 Department of Infectious Diseases, Internal Medicine, Hospital Universitario de Fuenlabrada, Fuenlabrada, Madrid Spain; 4 Centro de Investigación Biomédica en Red de Enfermedades Infecciosas (CIBERINFEC), Instituto de Salud Carlos III, Madrid, Spain; 5 Department of Microbiology, Hospital Universitario de Fuenlabrada, Fuenlabrada, Madrid Spain; 6 Faculty of Experimental Science, Universidad Francisco de Vitoria, Madrid, Spain; 7 HDT Bio, Seattle, WA USA; 8 Center for Global Infectious Disease Research, Seattle Children’s Research Institute, Seattle Children’s Hospital, Seattle, WA USA; 9 Department of Pediatrics, University of Washington School of Medicine, Seattle, WA USA; 10 Department of Global Health, University of Washington, Seattle, WA USA

**Keywords:** *Leishmania*, Recombinant antigens, Cellular response, Whole blood assay

## Abstract

**Background:**

The whole blood stimulation assay (WBA) is a valuable tool for detecting asymptomatic *Leishmania* infection and monitoring the treatment of visceral leishmaniasis (VL). This study sought to identify specific recombinant proteins to replace the nonspecific soluble *Leishmania* antigen in this assay, which could be useful for developing a standardized diagnostic test that complies with good manufacturing practice.

**Methods:**

Employing a cell lymphoproliferative assay, we here assessed the behaviour of 11 recombinant antigens in 61 subjects who had either been successfully treated for or had spontaneously recovered from *Leishmania infantum* infection. We then selected those antigens showing significant differences in immune cell stimulation indices and cytokine secretion between a responder and non-responder group, respectively, showing a cellular response to *L. infantum* or not. The three best candidate antigens, ΔCpB, NSC and ENSC, were then used in a WBA conducted on peripheral blood from 53 subjects stratified according to leishmaniasis status [cured VL, cured cutaneous leishmaniasis (CL), asymptomatic leishmaniasis (AS) and healthy controls].

**Results:**

ENSC was found to be the most effective antigen to detect cured VL by measuring specific IP-10 production (90% recognition) and TNF induced by ΔCpB to detect cured CL (71.4% recognition). Although the cytokines IL-2 and IP-10 elicited by NSC and ENSC were able to detect AS, this capacity was only moderate (60%).

**Conclusions:**

We propose that, once validated in larger studies, these GLP *Leishmania* antigens might help improve the accuracy of treatment monitoring and diagnosing cure.

**Graphical Abstract:**

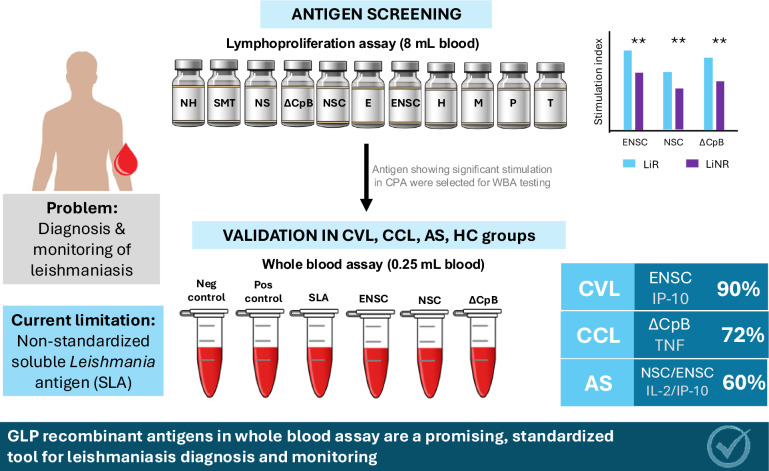

**Supplementary Information:**

The online version contains supplementary material available at 10.1186/s13071-025-07108-z.

## Background

Leishmaniasis is a neglected tropical disease caused by parasites of the *Leishmania* genus and transmitted by the bites of infected female phlebotomine sandflies. Globally distributed across 69 tropical and subtropical countries [1], leishmaniasis affects 0.27 million people each year, predominantly as cutaneous leishmaniasis (CL, 0.25 million cases), with 17.2 thousand visceral cases (VL). Despite a decline in incidence due to targeted control measures, 20–60% of infections remain asymptomatic, representing a hidden reservoir of infection that complicates disease control, surveillance and clinical management [1–4]. Effective clinical management and public health initiatives depend on robust biomarkers that accurately reflect infection status not only in asymptomatic infection but also in monitoring treatment success [4–6].

Although no human vaccines have been approved yet, significant progress has been made in understanding the immune mechanisms needed for a protective response against *Leishmania* [7–10]. Individuals with asymptomatic infection and those who have been successfully treated for VL and CL mount a strong specific Th1 (type-1 helper) cell response [11]. The cellular response associated with infection control leads to the specific proliferation of CD4^+^ T lymphocytes, which mostly produce interferon-γ (IFN-γ), tumour necrosis factor (TNF) and interleukin-2 (IL-2). These molecules activate the leishmanicidal function of macrophages, leading to the production of reactive oxygen and nitrogen intermediates, particularly nitric oxide and its derivates, which mediate intracellular parasite killing [12, 13]. IFN-γ-induced protein 10 (IP-10/CXCL10) is rapidly produced in response to TNF and IFN-α/β/γ and recruits Th1 cells to inflammatory sites. Additionally, the Th1 protective response is characterized by strong activation of CD8^+^ T cells, which kill infected macrophages by releasing perforins and granzymes [11, 14, 15]. Contrarily, IL-10 is an anti-inflammatory cytokine that inhibits the activity of Th1 cells, NK cells and macrophages, preventing pathogen clearance in VL.

To examine the cell activation response against *Leishmania*, ex vivo cell stimulation assays, such as the cell proliferation assay (CPA) or the whole blood assay (WBA), have been developed. These cellular tests can effectively identify asymptomatic subjects and support assessment of cure in individuals with VL, even in conditions of immunosuppression [16–18]. In the WBA, differences have been observed in the expression of a significant number of cytokines after soluble *Leishmania* antigen (SLA) stimulation among persons with asymptomatic leishmaniasis, cured VL and negative controls [6, 19]. Therefore, it is important to quantify the most relevant Th1 and Th2 cytokines after recombinant antigen stimulation.

One of the main limitations of the CPA and WBA assays is that they are both based on the use of SLA, which is not commercially available. It is therefore critical to find antigens capable of generating a Th1 cell response that can be produced under good manufacturing practice (GMP) conditions. The use of recombinant chimeric proteins would broaden the utility of these assays, mainly WBA, and offer easier and faster results in hospitals, health centres and point-of-care (PoC) scenarios [5, 6].

Recently, several recombinant antigens that give rise to a Th1 protective response against *Leishmania* have been described in mice, hamsters, dogs and in vitro cell cultures. Antigens such as tryparedoxin peroxidase (TRYP) [20, 21], *Leishmania*-activated C-kinase antigen (LACK) [20–22], histone H2B (H2B) [23–25], kinetoplastid membrane protein 11 (KMP-11) [26] and Leish-F3 and Leish-F3 + [27] have proved their capacity to activate T cells and/or produce an effective response in cured VL patients and asymptomatic subjects. Among them, Leish-F3 produced under GMP conditions has been reported safe and immunogenic for use in a clinical setting (Phase I trial) [28].

The aim of the present study was to examine the behaviour of several recombinant *Leishmania* antigens as potential candidates to replace SLA, mainly for use in WBA in field studies. Our ultimate goal was to develop a GMP assay capable of supporting the assessment of cured CL and VL patients as well as the detection of AS subjects. To this end, we first tested 11 recombinant antigens, components of chimeric antigens forming Leish-F3 and other promising constructs in a CPA assay using PBMC from 61 patients who had been successfully treated for *Leishmania infantum* infection. We then assessed the best performing candidates in a WBA, quantifying the production of IFN-γ, TNF, IL-2, IL-10, granzyme B and IP-10. Finding recombinant antigens that may elicit strong Th1 responses may help identify asymptomatic carriers and improve treatment outcomes. This information will help with patient monitoring, targeted interventions and reinforcement of leishmaniasis control programs in endemic areas.

## Methods

### Study population

Blood samples were taken from 94 volunteers, all aged > 18 years. All participants lived in an area with high *Leishmania infantum* infection prevalence (Fuenlabrada, Madrid) because of a past declared outbreak. Recruitment was conducted between 2015 and 2018, and the number of samples included was determined by volunteer and patient availability rather than a formal statistical power calculation. Of these subjects, 52 had no history of leishmaniasis: the healthy control group consisted of subjects with negative results in all tests for *Leishmania* (PCR, rK39-ICT, IFAT and CPA) (*n* = 14), and asymptomatic infection was defined as a negative result by molecular assay, but detectable cell stimulation and/or cytokine responses in CPA, in line with our previous works demonstrating the utility of this technique for this purpose [4, 19]. Participants were described as cured if their blood was parasite free after 3 months of treatment and also showed clinical recovery from the disease.

### Study design

All subjects who had recovered from *L. infantum* infection–either spontaneously or following treatment–were assigned to a responders group, LiR, of subjects showing a positive cellular response to SLA in the lymphoproliferation test (CPA) [stimulation index (SI) > 2.27] (*n* = 61) [19]. In contrast, non-responders (LiNR) were taken as those returning a negative result for this test (*n* = 33). The cut-off for a positive lymphoproliferative response was determined using a receiver-operating curve (ROC) [19].

This study was conducted in two stages. In the first stage, we screened participants for their cell response to 11 recombinant antigens by CPA. Then, some recombinant antigens were selected for further study based on significant differences in stimulation indices and cytokine production between the groups of responders and non-responders (LiR and LiNR). In the second study stage, the recombinant antigens selected were employed in a WBA conducted in four well-characterized groups of participants: (i) those cured of visceral leishmaniasis (CVL) (*n* = 10), (ii) those cured of cutaneous leishmaniasis (CCL) (*n* = 14), (iii) those with asymptomatic *Leishmania* infection (AS) (*n* = 15) and (iv) healthy control subjects (HC) (*n* = 14).

### DNA extraction

DNA was extracted from 200 µl of whole blood (CVL, AS and HC). For CCL, we used 200-µl skin biopsies placed in 400 µl of NET10 (10 mM NaCl, 10 mM EDTA, 10 mM Tris HCl), 40 µl of SDS (10%) and 2 µl of proteinase K and incubated overnight with agitation at 56 ºC. For DNA isolation, the phenol-chloroform method was employed with precipitation in ethanol [29]. Total DNA was resuspended in 200 µl of sterile distilled water and quantified using a UV-V ND-100 spectrophotometer (NanoDrop Technology, USA).

### *Leishmania*-nested PCR

DNA was subjected to nested PCR (Ln-PCR) using primer pairs that amplify the *Leishmania* small ribosomal subunit (SSUrRNA) [30] in a GenAmp PCR System 2700 thermocycler (Applied Biosystems, USA). The first round of reactions (30 cycles, annealing temperature 60 ºC) involved using primers R221 and R332. The amplicons were diluted 1/40 in distilled water, and 10 µl of this dilution was used in the second round of reactions employing primers R223 and R333 (30 cycles, annealing temperature 65 ºC). The amplicons were then visualized in 1.5% agarose gels in TAE buffer (Tris-acetate 0.04 mM, EDTA 1 μM, pH 8) stained with 0.02% GelRed (Biotium, USA) in a MiniBis-pro illuminator (DNR, Bio-imaging Systems, Israel). A result was classified as positive when amplicons of 358 bp were detected.

### rK39-immunochromatographic test (rK39-ICT)

The rK39-ICT (Kalazar Detect™ Rapid Test, InBios International Inc., Seattle, WA, USA) was performed according to the manufacturer’s instructions. Briefly, 25 µl of serum was added to the test strips along with the provided buffer solution in 2-ml Eppendorf tubes. Results were read after 10 min of incubation at room temperature. The strips were examined for the two bands (control and specific), indicating a positive result.

### Immunofluorescent antibody titres

Immunofluorescent antibody titres (IFAT) were determined in 1-μl serum samples according to the standard method [31]. The antigen was derived from *L. infantum* promastigotes (reference strain MHOM/FR/78/LEM-75) and antibody binding detected using fluorescein isothiocyanate-conjugated sheep anti-human IgG. A titre of ≥ 1/80 was considered the threshold for positivity.

### Antigens

Soluble *Leishmania* antigen (SLA) was prepared from a stationary phase *L. infantum* promastigote culture (JPC strain, MCAN/ES/98/LLM-722). The parasites were washed with phosphate-buffered saline (PBS) and then centrifuged at 1000 g for 20 min at 4 ºC. The supernatant was discarded and the pellet resuspended in lysis buffer (50 mM Tris/5 mM EDTA/HCl, pH 7). These samples were subjected to three cycles of freezing and thawing, followed by three probes (40 W for 20 s) and centrifugation at 27,000 g for 20 min at 4 ºC. To remove the antigens from the membrane, the supernatants were collected and then centrifuged at 100,000 g for 4 h at 4 ºC. Finally, the supernatants were divided into aliquots and stored at – 80 ºC until use. Protein contents were quantified using the BCA Protein Assay Kit (ThermoFisher Scientific, Spain).

All recombinant antigens were cloned and expressed in *E. coli* as previously described (Additional Table 1). NS antigen was produced under Good Manufacturing Practice (GMP) conditions, ensuring compliance with regulatory standards for clinical-grade materials. All other antigens were generated under Good Laboratory Practice (GLP) standards. Affinity-purified protein fractions were analysed by sodium dodecyl sulphate-polyacrylamide gel electrophoresis (SDS-PAGE) and quantified using the BCA Protein Assay Kit (ThermoFisher Scientific). Endotoxin levels measured in the Limulus Amebocyte Lysate QCL-1000^®^ assay (Lonza Inc., Basel, Switzerland) were all < 100 EU/mg protein.

We then went on to assess a wide range of *Leishmania* antigens in terms of their immunogenic properties across different host species. The NH antigen, a nucleoside hydrolase derived from *Leishmania donovani*, has been tested in humans as referenced [32, 33]. The SMT antigen (sterol-24-C-methyl-transferase) from *L. infantum* has been examined in both mice and humans [34–36]. The LeishF3 formulation (here designated NS) includes the NH36 antigen from *L. donovani* and SMT antigen from *L. infantum* and has been tested in mice, hamsters and humans [27, 28, 37, 38]. The ΔCpB antigen (delta cysteine protease B), also from *L. infantum*, has been analysed in mice [39]. The LeishF3 + (here designated NSC) formulation combines NH36, SMT and ΔCpB antigens and has been tested in mice, hamsters and humans [27, 28, 37]. Heat shock proteins (HSP) from *L. donovani* were included in our study as antigen E. Antigen ENSC comprises antigen E plus NH36, SMT and ΔCpB; its fusion was performed as in Coler et al. [28]. Other antigens employed here were H (histone H2B N-terminal), tested in mice and humans [23, 40]; M (malate dehydrogenase), tested in mice [23]; P (p21, a *Leishmania*-specific 21 kDa antigen of unknown function); and T (alpha tubulin), tested in mice [23].

### PBMC culture and cell proliferation assay (CPA)

Blood samples (10 ml) from all participants were collected into heparinized vials. From these samples, peripheral blood mononuclear cells (PBMCs) were isolated by Ficoll-Hypaque gradient (Lymphocytes Isolation Solution, Rafer, UK) and then resuspended in RPMI 1640 medium (Lonza, Sweden) supplemented with 10% fetal bovine serum (FBS) and 100 U/ml penicillin/streptomycin. Aliquots containing 2 × 10^5^ cells/well were placed in 96-well plates and cultured for 5 days in supplemented RPMI 1640 medium, either alone as a negative control or with the addition of *Leishmania* antigens (10 µg/ml for SLA or recombinant antigens) at 37 ºC in a 5% CO_2_ incubator. Due to the limitations of PBMC quantities, the 11 antigens were not tested in all the subjects enrolled; rather, they were sequentially tested in a minimum of 20 participants per antigen. In the last 24 h of incubation, 5-bromo-2 deoxyuridine (BrdU) was added to analyse lymphocyte proliferation using the BrdU Cell Proliferation Assay Kit (Merck, Roche Life Science Products, Germany) according to the manufacturer’s instructions. Results were expressed as the stimulation index (SI) (absorbance mean of stimulated cells/absorbance mean of unstimulated cells). The supernatants were collected and stored at – 20 ºC for later analyte analysis.

### Whole blood stimulation assay (WBA)

For each participant, 250 μl of whole blood was placed in each of three tubes containing the following: empty (negative control); 10 μg/ml of SLA, and 5 µg/ml phytohemagglutinin (PHA, ThermoFisher Scientific) (positive control). Additionally, three tubes containing 10 μg/ml as the final concentration of each recombinant antigen were assayed. This concentration was previously optimized. All tubes were incubated at 37 °C for 24 h. After centrifugation at 2,000 g for 10 min, plasma samples were removed and kept at − 20 °C for later cytokine analysis.

### Quantifying cytokine expression in supernatants and plasma

Expression levels of interferon-gamma (IFN-γ), tumour necrosis factor-alpha (TNF) and interleukin 10 (IL-10) were determined in supernatant and plasma samples, and interleukin 2 (IL-2), granzyme B and interferon-inducible protein 10 (IP*-*10 or CXCL10) in plasma. Measurements were performed by flow cytometry using the Cytometric Bead Array Human Flex Set (Beckton & Dickinson Bioscience, USA) following the manufacturer's instructions. Briefly, 50 μl of the supernatant/plasma of each participant was incubated for 1 h at room temperature with 50 μl of capture beads. After incubation, 50 μl of the detection antibody was added and the mixture was incubated for an additional 2 h at room temperature. Data acquisition was performed using a FACSCalibur^®^ cytometer, and analysis was conducted with FCAP Array software Version 3.0 (Beckton & Dickinson Bioscience, USA). Cytokine and chemokine concentrations (expressed in pg/ml) in response to SLA- or recombinant antigen stimulation were determined by subtracting background expression levels measured in the negative control samples.

### Statistical analysis

All calculations were performed using GraphPad Prism 9.0 software (GraphPad Software, USA). Data were first assessed for normality. Comparisons between groups were performed using multiple pairwise Mann-Whitney tests. To account for the increased risk of type I error due to multiple comparisons, *P*-values were adjusted using the Holm-Bonferroni method. Data were presented as the median and interquartile range (IQR), and *P* < 0.05 was considered statistically significant. The cut-off was determined by calculating the area under the curve (AUC).

## Results

### Clinical characteristics of participants

Samples from 94 subjects were subjected to CPA employing recombinant antigens. Of these, 61 without symptoms showed a positive cell response (LiR) indicating controlled *L. infantum* infection, either spontaneously or following treatment. None of the subjects tested were PCR positive for the parasite, and 20% of individuals with CVL maintained a humoral response after treatment as measured by IFAT and rk39-ICT (Additional Table 2). In contrast, 33 subjects showed no cellular response and tested negative for *L. infantum* in molecular and serological tests (LiNR).

For the WBA recombinant antigen validation stage conducted in 53 subjects, we used 10 blood samples from subjects with CVL, 14 from those with CCL, 15 from AS and 14 from HC. There was no detectable parasite load in any of the groups. *Leishmania*-specific antibodies were present in 20% of participants in the CVL group and 5.3% in the AS group.

### ΔCpB, NSC and ENSC antigens elicit a specific lymphoproliferative response plus IFN-γ/TNF secretion in PBMC

**Fig. 1:**
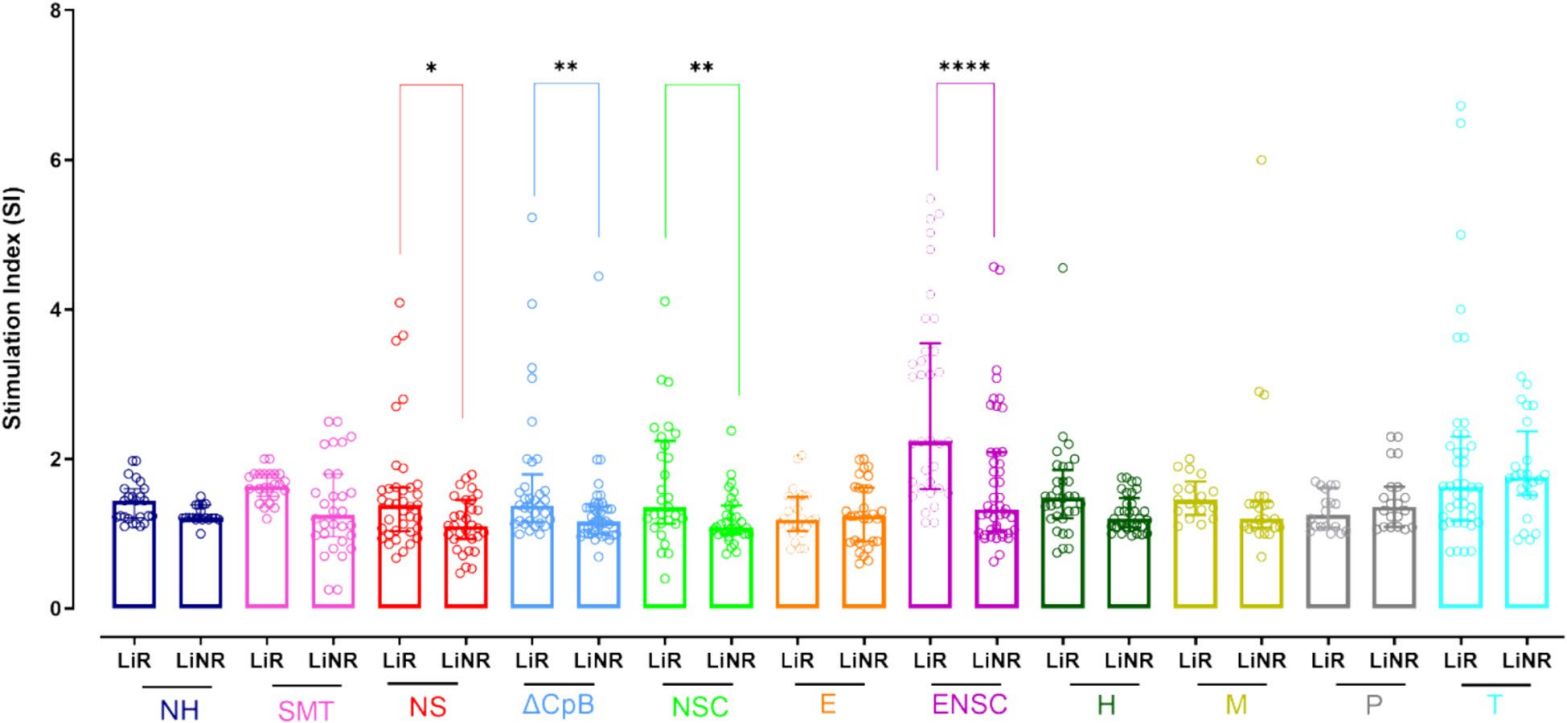
Stimulation indices recorded in response to several recombinant antigens (NH, SMT, NS, ΔCpB, NSC, E, ENSC, H, M, P, T) in the presence of PBMC from individuals showing a cellular response to *Leishmania* (LiR) (*n* = 30) and those showing no such response (LiNR) (*n* = 25). Data are presented in box-and-whisker plots in which boxes indicate the interquartile range (IQR), and individual dots represent sample values. Data were analysed using the Mann-Whitney U test with Holm-Bonferroni correction for multiple comparisions. Significance is indicated by asterisks: *P < 0.05; **P < 0.01; ****P < 0.0001

We observed that recombinant NS, ΔCpB, NSC and ENSC antigens prompted a significantly greater lymphoproliferative response (CPA) in LiR PBMC compared to LiNR PBMC (NS *P* = 0.0262; ΔCpB *P* = 0.0027; NSC *P* = 0.0085; ENSC < 0.0001) (Fig. 1).

In the PBMC supernatants from LiR participants, we detected significant IFN-γ and TNF production along with low IL-10 levels in response to stimulation with ΔCpB, NSC and ENSC antigens (Additional Figs. 1, 2 and 3). Hence, both intense lymphoproliferation and IFN-γ and TNF secretion were used as the criteria to select NSC, ENSC and ΔCpB for further WBA analysis.

### Cured and asymptomatic patients mount a Th1 cellular response when exposed to recombinant antigens in a WBA

**Fig. 2:**
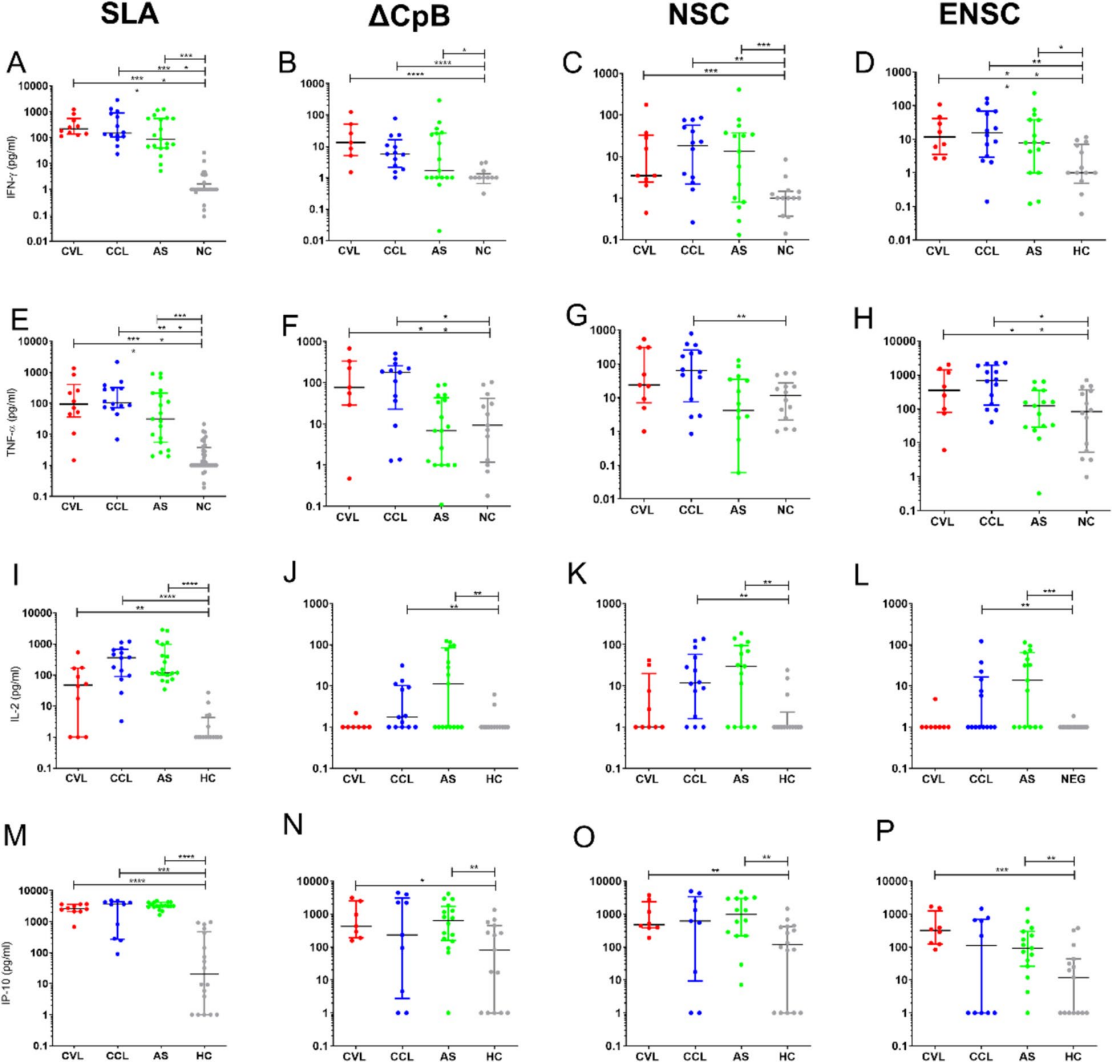
Cytokines produced in the whole blood response to SLA and recombinant antigens. Production of IFN-γ, TNF, IL-2 and IP-10 after stimulation of whole blood with SLA, ΔCpB, NSC and ENSC after 24 h of incubation in subjects cured of visceral leishmaniasis (CVL; *n* = 10), subjects cured of cutaneous leishmaniasis (CCL; *n* = 14), asymptomatic subjects (AS; *n* = 15) and healthy controls (HC; *n* = 14). Horizontal bars represent the median concentration. Data were analysed using the Mann-Whitney U test with Holm-Bonferroni correction for multiple comparisions. *P < 0.05, **P < 0.01, ***P < 0.001, ****P < 0.0001

The three most promising candidates, ΔCpB, NSC and ENSC, were then used to assess cellular recognition after 24 h of stimulation in a WBA conducted in our different clinically characterized participant groups, CCL, CVL, AS and HC. The resultant data revealed that patients cured of leishmaniasis and asymptomatic subjects produced higher levels of IFN-γ after stimulation with ΔCpB, NSC or ENSC compared to levels produced in healthy control individuals (Fig. 2). Specifically, in the CVL group, median (IQR) levels of IFN-γ after stimulation with ΔCpB were 13.18 (5.05–50.89) pg/ml, and after stimulation with ENSC, they were 11.72 (3.51–41.40) pg/ml. In CCL, median (IQR) levels of IFN-γ after stimulation were 5.71 pg/ml (2.15–16.36) for ΔCpB, 18.30 pg/ml (2.43–32.76) for NSC and 15.47 pg/ml (3.51–41.40) for ENSC. Finally, in the AS group, IQR levels of IFN-γ in response to NSC stimulation were 13.30 (0.80–36.98) pg/ml (Fig. 2B–D).

Significantly higher levels of TNF were found after stimulation with ΔCpB (77.57 pg/ml) and ENSC (350.70 pg/ml) in CVL patients compared to healthy controls (9.25 pg/ml and 82.77 pg/ml, respectively) (Fig. 2E–H). In CCL patients, this cytokine was secreted in significantly greater amounts after stimulation with all three recombinant antigens, ΔCpB (*P* = 0.0061), ENSC (*P* = 0.0025) and NSC (*P* = 0.0081), in relation to the HC group (Table 1).

**Table 1:** Medians (pg/ml) and interquartile ranges (IQR) of cytokines produced in plasma stimulated with soluble *Leishmania* antigen (SLA) and recombinant antigens

Groups	SLA	ΔCpB	NSC	ENSC
IFN-γ				
CVL	215.1 (137.5–547.6)	13.18 (5.05–50.89)	3.47 (2.43–32.56)	11.72 (3.51–41.40)
CCL	148.0 (104.8–900.9)	5.71 (2.15–16.36)	18.30 (2.43–32.76)	15.47 (3.51–41.40)
AS	87.64 (40.31–551.1)	1.68 (0–26.57)	13.30 (0.80–36.98)	7.84 (0.00–37.67)
HC	0.00 (0.00–1.63)	0.00 (0.00–1.33)	0.25 (0.00–1.45)	0.40 (0.00–6.99)
TNF				
CVL	93.65 (36.45–403.2)	77.57 (28.90–332.20)	23.63 (7.05–541.1)	350.70 (79.28–1418)
CCL	105.0 (72.23–320.4)	178.10 (22.72–257.40)	62.66 (7.48–255.4)	687.70 (126.70–1932)
AS	31.11 (5.50–213.1)	6.81 (0.00–43.16)	4.17 (0.06–34.61)	122.0 (28.81–634.5)
HC	0.00 (0.00–3.760)	9.25 (1.17–41.84)	11.46 (2.16–27.64)	82.77 (5.15–362.10)
IL-2				
CVL	47.64 (0.00–166.3)	0.00 (0.00–1.00)	0.00 (0.00–19.85)	0.00 (0.00–1.00)
CCL	363.0 (90.14–667.8)	1.74 (0.00–10.18)	11.79 (1.59–58.39)	0.00 (0.00–16.35)
AS	118.4 (98.84–975.0)	11.28 (0.00–83.85)	29.96 (0.00–93.91	13.68 (0.00–64.71)
HC	0.00 (0.00–4.27)	0.00 (0.00–1.00)	0.00 (0.00–2.29)	0.00 (0.00–1.00)
IP-10				
CVL	2606 (2117–3626)	429.2 (189.20–2478)	478.3 (379.30–2369)	311.8 (123.6–1232)
CCL	3598 (271.3–4344)	231.2 (2.75–3090)	611.9 (9.25–3350)	109.1 (0.00–677.0)
AS	3183 (2973–4171)	635 (156.7–1679)	967.60 (216.90–2948)	91.43 (25.82–293)
HC	20.6 (0.00–471.40)	81.60 (0.00–451.3)	116.8 (0.00–412.4)	11.83 (0.00–43.11)
IL-10				
CVL	4.86 (2.28–33.33)	20.74 (1.45–147.50)	1.25 (0.33–87.24)	170.0 (19.77–466.6)
CCL	35.33 (2.63–313.0)	113.40 (7.37–357.1)	31.99 (3.19–265.30)	376.20 (57.33–1624)
AS	0.00 (0.00–1.00)	1.00 (0.85–2.43)	1.00 (0.00–2.27)	47.84 (7.99–160.5)
HC	0.00 (0.00–1.00)	1.00 (0.85–5.01)	1.35 (0.92–3.44)	84.69 (1.70–183.4)
Granzyme B				
CVL	705.0 (452.8–1007)	97.47 (61.44–271.4)	214.30 (75.56–282.60)	283.9 (182.9–404.6)
CCL	461.4 (157.6–5565)	96.69 (31.22–591.3)	99.99 (22.79–461.00)	423.00 (128.20–1915)
AS	51.56 (15.17–486.8)	0.00 (0.00–25.31)	0.00 (0.00–39.58)	59.51 (6.16–581.2)
HC	10.81 (1.17–37.75)	42.54 (0.00–168.8)	2.83 (0.00–60.33)	157.90 (9.42–474.7)

Data were collected from cured patients with visceral or cutaneous leishmaniasis, asymptomatic individuals and healthy controls

*CVL* cured visceral leishmaniasis, *CCL* cured cutaneous leishmaniasis, *AS* asymptomatic *Leishmania* infection, *HC* healthy controls

Our data for the regulatory cytokine IL-10 indicated higher than control levels produced in response to the ΔCpB antigen in all patients cured of leishmaniasis (Additional Fig. 4B). Additionally, ENSC and NSC led to higher levels of this cytokine in CCL patients (Table 1) (Additional Fig. 4C–D).

Our results for the cytokine IL-2 revealed its significantly greater production in the AS compared to HC group after stimulation with all three recombinant antigens (all *P* < 0.01) (Fig. 2I–L). A similar pattern was observed in the CCL group. Furthermore, after stimulation with ΔCpB, NSC and ENSC, CVL patients showed secretion of chemokine IP-10 (Table 1). The AS group showed median (IQR) IP-10 values of 635 pg/ml (156.7–1679; *P* = 0.0086) for ΔCpB, 967.60 pg/ml (216.90–2948; *P* = 0.0068) for NSC and 91.43 pg/ml (25.82–293; *P* = 0.0062) for ENSC (Fig. 2M–P). Moreover, NSC significantly induced the production of granzyme B in CVL and CCL patients (Table 1) (Additional Fig. 4G). Similarly, ENSC produced greater amounts of granzyme B in CCL patients compared to healthy controls (Additional Fig. 4H).

### Best recombinant antigens in a WBA for CVL, CCL and AS

**Fig. 3:**
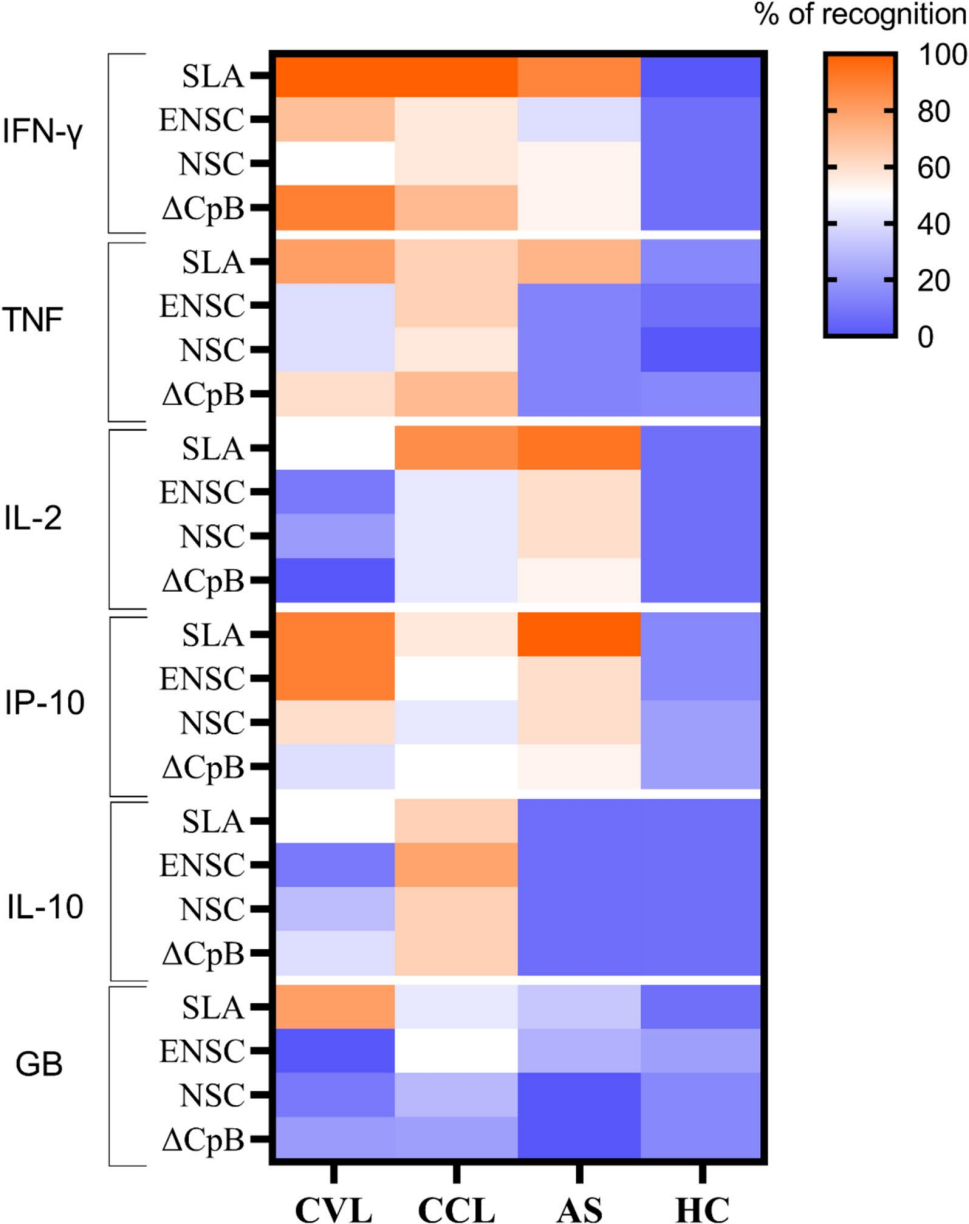
Heatmap showing percentages of immune recognition elicited by GLP-grade recombinant *Leishmania* antigens across four clinical subsets of participants. In this test, whole blood from subjects cured of visceral leishmaniasis (CVL), those cured of cutaneous leishmaniasis (CCL), asymptomatic subjects (AS) and healthy controls (HC) was stimulated with recombinant antigens. The heatmap displays the percentages of individuals in each group showing a positive cytokine response to each antigen (rows). Warmer colours indicate higher recognition rates and cooler colours a lower recognition capacity

Specific recombinant antigens against *Leishmania* were identified based on patient recognition and cytokine production. Figure 3 presents antigen recognition percentages and corresponding cytokine production based on cut-offs for each antigen and analyte. The full diagnostic performance, including AUC, sensitivity, specificity and cut-off values for each cytokine and chemokine, is provided in additional Table 3. Some discrete production of Granzyme B and IP-10 was observed in healthy individuals, likely because of innate activation of various cell types present in whole blood. Focusing on the characterization of antigen-specific cytokine responses to *Leishmania*, our study identified ΔCpB, NSC and ENSC as recall antigens of a Th1 cellular response after restimulation in the groups CCL, AS and CVL.

In CVL patients, we found that WBA using the ENSC antigen along with IP-10 quantification, or the ΔCpB antigen and IFN-γ quantification, was the best combination to measure specific cellular responses (90% compared to SLA). If we also consider the amounts of cytokine produced after stimulation of whole blood from CVL with the recombinant antigens in relation to SLA, the best antigen/cytokine combination for this patient subset was ENSC/IP-10 [ENSC 311.8 pg/ml (123.6–1232), vs SLA 2606 pg/ml (2117–3626)]. As indicated in Table 1, relative to SLA stimulation, ENSC antigen stimulation plus IP-10 quantification resulted in eightfold lower cytokine levels, while ΔCpB antigen stimulation and IFN-γ quantification led to levels that were 16-fold lower.

For CCL patients, the most suitable combination was ΔCpB antigen stimulation plus IFN-γ or TNF measurement (71.4% compared to SLA). However, ΔCpB stimulation and IFN-γ measurement yielded 37-fold lower amounts of cytokine compared to SLA, while ΔCpB stimulation combined with TNF measurement determined similar cytokine production to SLA. For this reason, the best antigen to identify cases of CCL would be ΔCpB combined with TNF measurement [ΔCpB 178.10 pg/ml (22.72–257.40) vs SLA 105.0 pg/ml (72.23–320.4)].

Finally, to identify subjects with asymptomatic *Leishmania* infection, NSC and ENSC emerged as the best antigens for the WBA along with IL-2 or IP-10 assessment, although their capacity to detect these subjects was only moderate (60%) (Additional Table 4).

Our results regarding the detection capacity of the antigens to use with granzyme B were low for all the participant groups examined here (Additional Table 4). In addition, granzyme B and IP-10 showed some unspecificity in that these analytes were detected in the HC group (Fig. 3, Additional Table 4).

For comparative purposes, IL-10 data are also provided (Table 1, Additional Table 4). Due its regulatory nature and role in the pathogenesis of leishmaniasis, we did not expect this cytokine to elicit a positive cellular response to *Leishmania*.

## Discussion

To examine the immune cell response to *Leishmania*, the in vitro whole blood stimulation technique has proven faster and easier than CPA. Several studies have also confirmed that WBA is an effective tool to detect cured or asymptomatic individuals in the field [6, 19, 20]. Currently, the antigen employed in both tests is the soluble *Leishmania* antigen, whose production has not yet been undertaken under GMP conditions. This determines a need to search for GMP-grade recombinant *Leishmania* antigens such as those used in QuantiFERON's commercial test for tuberculosis and cytomegalovirus.

The setting for the present study was an *L. infantum* endemic area. Here, the fusion protein ENSC emerged as an effective antigen to detect CVL through the cell production of the cytokines IFN-γ (70% recognition) and IP-10 (90% recognition). In effect, quantification of IP-10 produced in response to ENSC in the WBA seems the most promising method, as the high levels of this chemokine observed could be easily detected using a rapid immunochromatographic test, although this has yet to be developed. As widely established, IP-10 is critical to induce cellular immunity following vaccination against *L. donovani* [41] and shows a sharp increase in patients cured of VL [6, 42]. ENSC is the result of fusion of heat shock proteins (HSP) and Leish-F3 +. Although in our study it was not possible to detect a response to the HSP antigen E in a high number of subjects, in general HSPs have been found to enhance the immunogenicity of weak antigens and stimulate antigen presenting cells [43]. In effect, while we noted poor responses to ΔCpB and NSC antigens (as previously described by our group [27]), antigenicity was enhanced when HSP antigen E was added to Leish-F3 +. The latter has been described as capable of inducing a Th1 response in mice and humans [28] and protecting against *Leishmania* infection in mice [37]. In addition, this antigen was also found to protect hamsters infected with *L. infantum* when formulated with KMP-11 and LJL143 antigens in virosomes and administered along with GLA-SE adjuvant [44]. In the present study, the ΔCpB protein was also found effective in detecting CVL via IFN-γ quantification (90% recognition). In similar studies, VL patients from a region endemic for *L. donovani* were found to have CD4^+^ T cells producing specific IFN-γ, IL-2 and/or TNF [12/20 (60%), 11/20 (55%) and 17/20 (85%), respectively] after ΔCpB stimulation. Moreover, *Leishmania* cysteine protease B (CpB) has shown promise as a candidate VL vaccine antigen in mouse and dog infection models [45, 46]. The truncated recombinant variant of CpB (ΔCpB), devoid of potential self-reactive epitopes, has also displayed T-cell antigenicity and its potential use in a vaccine [38].

In this line of research, in response to WBA stimulation, the M18 antigen, an amastin-like protein, elicited the production of IFN-γ in 90% (9/10) of cured VL cases caused by *L. infantum* in Brazil [47]. Once isolated under GMP conditions, future studies should test this antigen in a larger number of control individuals to further address the unspecificity observed in that work [47] (1/4, 25%). Similarly in India [20], the stimulation of whole blood with R71 and N52 over 24 h led to IFN-γ production in a high percentage of individuals cured of VL caused by *L. donovani*. However, the amount of IFN-γ detected in most subjects was low, and it could be that IP-10 levels varied more from those determined in control samples, as noted here.

Pending validation studies, these findings provide direction for studies designed to assess the use of the WBA technique in combination with these specific recombinant antigens to confirm cured VL in regions endemic for *L. infantum* and *L. donovani* in settings of PoC.

In cured CL patients, we found IFN-γ secretion in response to LEISH-F3 in supernatants from PBMC. Similar results have been described in CL patients infected with *Leishmania major* and *L. tropica* [48]. Although we found some recognition of NSC- and ENSC-associated antigenic variations in the WBA, this was only partial (57.1–64.3%). However, using ΔCpB in WBA followed by the detection of IFN-γ or TNF, we were able to identify 71.4% of these CL patients. As far as we know, this is the first report of the determination of this protein in cured CL patients. Indeed, we propose this tool could have useful field applications to distinguish CL from other skin diseases with similar clinical symptoms. Conservation values of this antigen are 99% for *Leishmania donovani*, 68% for *L. major*, 73% for *L. mexicana* and 83% for *L. tropica* [38]. Consequently, research in other endemic areas than those of *L. infantum* is key to test whether ΔCpB is recognized by other species causing CL.

In endemic areas, large numbers of inhabitants are infected with *Leishmania* spp. yet never develop signs or symptoms of VL [3]. These AS individuals are easily detected in a WBA employing SLA and quantifying either IL-2 in regions endemic for *L. infantum* or monokine induced by IFN-γ (MIG) in areas harbouring *L. donovani* [6, 19]. Using NSC or ENSC and quantifying IL-2 or IP-10, we here detected 60% of AS subjects enrolled in our study from an endemic *L. infantum* area, with particularly high levels observed of IP-10. For CVL, R71 antigen was found to elicit IFN-γ responses in AS subjects from an *L. donovani* endemic area [20]. Unfortunately, however, the levels of this cytokine were quite low and close to control levels.

To better understand the findings, it is important to acknowledge the limitations of the current study. First, because sample quantities were limited, cytokines and chemokine responses were measured at a single time point, making it unable to evaluate intra-individual dynamics across time. Second, CPA was employed in part to define asymptomatic *Leishmania* infection [4, 20, 47], which may lead to incorporation bias if the same assay is later used to assess antigen-specific responses. The effectiveness of these recombinant antigens should be confirmed in areas where various *Leishmania* species are prevalent, as the study was geographically limited to *Leishmania infantum* infections in Spain. Third, while endotoxin levels were low (< 0.2 EU/well for CPA and < 0.5 EU/tube for WBA at working concentrations), the absence of irrelevant recombinant protein or polymyxin B controls remains a limitation, as residual contaminants cannot be completely excluded. Fourth, host-related factors such as genetic background, prior pathogen exposures and comorbidities may also shape immune responses and influence assay outcomes, limiting how widely the finding can be applied.

Given these limitations, the study shows that conserved parasite proteins can trigger measurable and unique cytokine responses. This suggest they could be useful for broader diagnostic purposes. To reduce uncertainty and improve standards for identification of asymptomatic infection and tracking treatment patients, future research should include studies across different endemic areas and at multiple time points during treatment.

## Conclusions

This study identifies *Leishmania*-derived T cell-stimulating antigens in subjects mounting a protective immune response against leishmaniasis. The quantification of IP-10 after the field WBA assay using ENSC was able to detect 90% of cases of CVL, while TNF measurement following ΔCpB stimulation identified 71.4% of those of CCL. These antigens, produced under GLP conditions, could help improve the accuracy of cure assessment tests pending their validation in larger studies. In addition, we anticipate that antigens like ENSC or ΔCpB will be candidates for inclusion in a subunit vaccine against leishmaniasis.

## Supplementary Information

Additional file 1. Clinical characteristics of the study population; cut-offs of SLA and recombinant antigens used to identify CVL, CCL and asymptomatic individuals; recognition percentages of subjects after stimulation of whole blood with the soluble *Leishmania* and recombinant antigens.

Additional file 2. IFN-γ, TNF and IL-10 levels secreted by PBMC after stimulation with NS, ΔCpB, NSC, E, ENSC or H antigens; cytokine production in the whole blood response to SLA and recombinant antigens.

## Data Availability

All data generated or analysed during this study are included in this published article.
