# Supplementary material for: Diagnostic potential of GLP recombinant antigens in whole blood assays for Leishmania infantum infection
Source: Parasit Vectors. 2025 Nov 21;18:475. doi: 10.1186/s13071-025-07108-z (PMC12639648; doi:10.1186/s13071-025-07108-z)
Supplement: Supplementary file 1 — Additional file 1. Clinical characteristics of the study population; cut-offs of SLA and recombinant antigens used to identify CVL, CCL and asymptomatic individuals; recognition percentages of subjects after stimulation of whole blood with the soluble Leishmania and recombinant antigens. [file 13071_2025_7108_MOESM1_ESM.docx]

**Additional Figure 1.** IFN-γ levels secreted by PBMC from LiR (*Leishmania infantum* responders) and LiRN (*Leishmania infantum* non responders) after stimulation with NS, ΔCpB, NSC, E, ENSC or H antigens. Data are represented as box-and-whisker plots, in which boxes indicate the interquartile range (IQR), and individual dots represent sample values. Data were analyzed using the Mann-Whitney U test. ***p<0.001; ****p<0.0001.

**Additional Figure 2.** TNF levels secreted by PBMC from LiR and LiRN after stimulation with NS, C, NSC, E, ENSC or H antigens. Data are represented as box-and-whisker plots, in which boxes indicate the interquartile range (IQR), and individual dots represent sample values. Data were analyzed using the Mann-Whitney U test. *p< 0.05; **p< 0.01; ***p< 0.001.

**Additional Figure 3.** IL-10 levels secreted by PBMC from LiR and LiRN after stimulation with NS, C, NSC, E, ENSC or H antigens. Data are represented as box-and-whisker plots, in which boxes indicate the interquartile range (IQR), and individual dots represent sample values. Data were analyzed using the Mann-Whitney U test. *p< 0.05; **p< 0.01; ***p< 0.001.


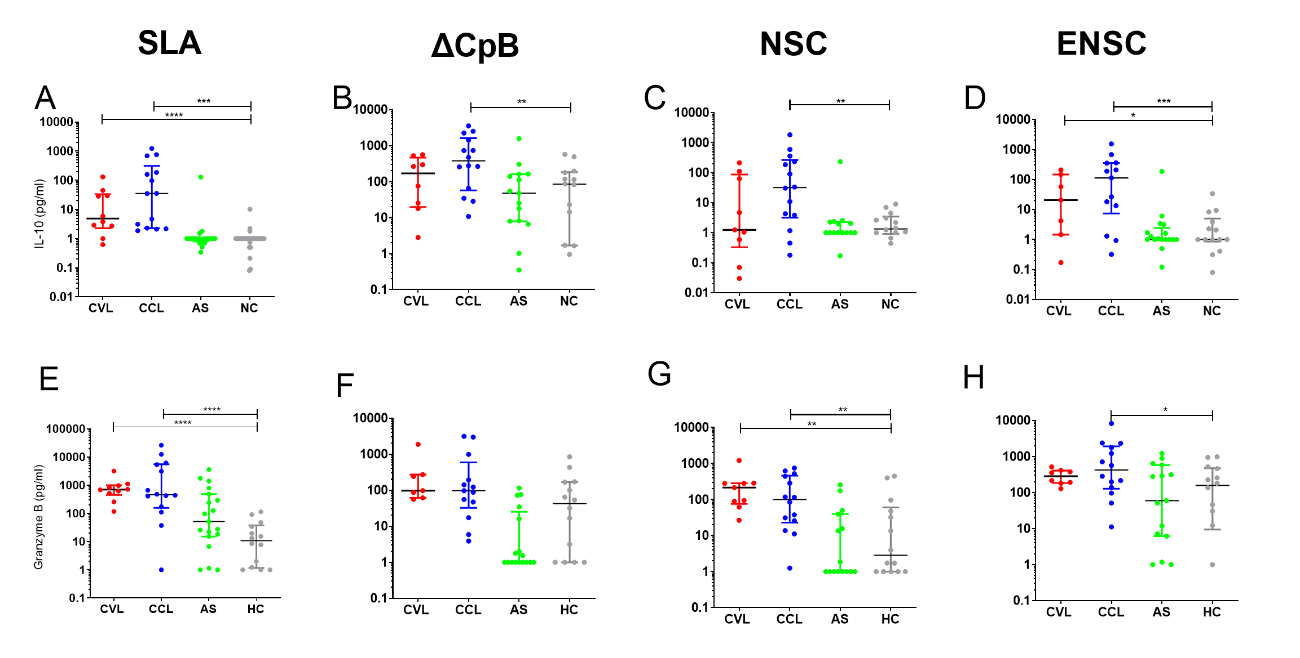


**Additional Figure 4.** Cytokine production in the whole blood response to SLA and recombinant antigens. Production of IL-10 and granzyme B after stimulation of whole blood with SLA, ΔCpB, NSC or ENSC after 24 h of incubation in subjects cured of visceral leishmaniasis (CVL; n=10), subjects cured of cutaneous leishmaniasis (CCL; n=14), asymptomatic subjects (AS; n=15), and healthy controls (HC; n=14). Horizontal bars represent the median concentration. The Mann~~-~~Whitney U test was used to compare medians. *p< 0.05, **p< 0.01, ***p< 0.001, ****p< 0.0001.
