# Supplementary material for: Diagnostic potential of GLP recombinant antigens in whole blood assays for Leishmania infantum infection
Source: Parasit Vectors. 2025 Nov 21;18:475. doi: 10.1186/s13071-025-07108-z (PMC12639648; doi:10.1186/s13071-025-07108-z)
Supplement: Supplementary file 2 — Additional file 2. IFN-γ, TNF and IL-10 levels secreted by PBMC after stimulation with NS, ΔCpB, NSC, E, ENSC or H antigens; cytokine production in the whole blood response to SLA and recombinant antigens. [file 13071_2025_7108_MOESM2_ESM.docx]

**Additional Table 1**. Recombinant antigens evaluated in leishmaniasis patient samples. The figure summarizes the key features of each antigen, including their name, molecular composition, production source, database accession number, and molecular size.

| **Antigen** | **Composition** | **Source** | **Accesion number** **Genbank** | **Fragment expressed** | **Size (kDa)** | **References** |
| --- | --- | --- | --- | --- | --- | --- |
| NH | Nucleoside hydrolase | *L. donovani* | XP_003860171.1 | full length | 36 | (1) |
| SMT | Sterol-24-c-methyl-transferase | *L. infantum* | XP_001469832.1 | full length | 40 | (1, 2) |
| NS = LeishF3 | NH: Nucleoside hydrolase | *L. donovani* | XP_003860171.1 | full length | 74 | (3) |
|  | SMT: Sterol 24-C-methyl transferase | *L. infantum* | XP_001469832.1 |  |  |  |
| ΔCpB | Delta cysteine protease B | *L. infantum* | XP_001463206.1 | full length | 34 | (4) |
| NSC = LeishF3+ | NH: Nucleoside hydrolase | *L. donovani* | XP_003860171.1 | full length | 106 | (3) |
|  | SMT: Sterol 24-C-methyl transferase | *L. infantum* | XP_001469832.1 |  |  |  |
|  | Δ CpB: Delta Cysteine Proteinase B | *L. infantum* | XP_001463206.1 |  |  |  |
| E | Heat Shock Protein 8E | *L. donovani* | AYU81047.1 | ∆ 509-661 | 17 | (2) |
| ENSC | E : Heat Shock Protein 8E | *L. donovani* | AYU81047.1 | full length  E: ∆ 509-661 | 123 |  |
|  | NH: Nucleoside hydrolase | *L. donovani* | XP_003860171.1 |  |  |  |
|  | SMT: Sterol 24-C-methyl transferase | *L. infantum* | XP_001469832.1 |  |  |  |
|  | Δ CpB: Delta Cysteine Proteinase B | *L. infantum* | XP_001463206.1 |  |  |  |
| H | Histone H2B n-term (H2Bn) | *L. major* | XP_001682580.1 | full length | 12 | (5) |
| M | Malate dehydrogenase (MDH) | *L. major* | XP_001682648.1 | full length | 33 | (6) |
| P | p21: *Leishmania* specific 21 kDa antigen | *L. major* | XP_003722922.1 | full length | 22 | (6) |
| T | alpha tubulin (aT) | *L. major* | XP_001681772.1 | full length | 50 | (6) |

**Additional Table 2**. Clinical characteristics of the study population.

| Characteristic | First study stage - CPA | |  | Second study stage -  WBA | | | |
| --- | --- | --- | --- | --- | --- | --- | --- |
|  | LiR | LiNR |  | CVL | CCL | AS | HC |
| Number of subjects | 61 | 33 |  | 10 | 14 | 15 | 14 |
| Median age (years) | 50 | 43 |  | 42 | 60 | 42 | 43 |
| Gender | | | | | | | |
| % Males | 64 | 58 |  | 30 | 36 | 89 | 64 |
| PCR (%) | | | | | | | |
| positive | 0 | 0 |  | 0 | 0 | 0 | 0 |
| IFAT (%) | | | | | | | |
| positive | 20 | 0 |  | 20 | 0 | 5.3 | 0 |
| rK39-ICT (%) | | | | | | | |
| positive | 20 | 0 |  | 20 | 0 | 5.3 | 0 |
| Treatment | | | | | | | |
| Amphotericin B liposomal | 41 | - |  | 100 |  | - | - |
| Meglumine antimoniate | - | - |  | 0 | 12 | - | - |

LiR: responders group; LiNR: non-responders group; CVL: cured VL; CCL: cured CL; AS: asymptomatic *Leishmania* infection; HC: healthy controls. PCR: polymerase chain reaction; IFAT: immunofluorescent antibody titre; rK39-ICT: rK39- immunochromatographic test.

**Additional Table 3.** Performance metrics for each recombinant antigen across all cytokine and chemokine readouts. For every antigen–analyte combination, the table shows area under the curve (AUC), sensitivity (Se), specificity (Sp), and cut-off values.

| **Analytes** | **Antigen** | **AUC** | **Se% (95% CI)** | **Sp (95% CI)** | **Cut-of (pg/mL)** |
| --- | --- | --- | --- | --- | --- |
| IFN-γ | ENSC | 0.7284 | 64 (50.14 - 75.68) | 70 (56.25 - 80.90) | 11.36 |
|  | NSC | 0.7398 | 62 (47.96 - 73.53) | 69 (55.73 - 80.09) | 5.82 |
|  | ∆CpB | 0.7700 | 58 (44.28 – 63.61) | 81 (68.06 – 89.81) | 2.99 |
| TNF | ENSC | 0.6794 | 36 (23.63 – 49.01) | 86 (74.28 -93.19) | 411.9 |
|  | NSC | 0.6152 | 74 (60.45 – 84.13) | 48 (34.80 - 61.49) | 55.52 |
|  | ∆CpB | 0.5974 | 70 (55.19 - 80.92) | 46 (32.15 -59.82) | 70.03 |
| IL-2 | ENSC | 0.7809 | 69 (54.97 – 79.67) | 73 (59.05-82.89) | 5.31 |
|  | NSC | 0.7401 | 65 (50.99 - 76.37) | 73 (59.05 - 82.89) | 18.66 |
|  | ∆CpB | 0.7936 | 73 (59.00 - 83.43) | 73 (59.00 - 83.43) | 7.14 |
| IP-10/ CXCL10 | ENSC | 0.8010 | 53 (39.23 – 66.67) | 76 (62.78 – 84.40) | 87.22 |
|  | NSC | 0.6671 | 63 (48.87 - 76.22) | 71 (55.78 - 81.84) | 657.2 |
|  | ∆CpB | 0.7296 | 67 (51.55 - 78.99) | 74 (58.93 - 84.70) | 478.2 |
| IL-10 | ENSC | 0.8226 | 20 (11.02 – 32.46) | 94 (84.08 - 98.40) | 508.2 |
|  | NSC | 0.5423 | 25 (15.23 - 39.21) | 77 (63.87 - 86.28) | 10.59 |
|  | ∆CpB | 0.5959 | 25 (14.92 - 38.78) | 77 (63.46 - 86.69) | 19.47 |
| Granzyme B | ENSC | 0.5060 | 73 (59.05 – 82.89) | 37 (25.32 – 50.97) | 511.3 |
|  | NSC | 0.6860 | 87 (74.73 – 93.32) | 47 (33.34 – 59.66) | 345.2 |
|  | ∆CpB | 0.6734 | 88 (75.30 – 94.12) | 48 (34.46 – 61.67) | 351.3 |

**Additional Table 4.** Recognition percentages of subjects cured of visceral leishmaniasis, cutaneous leishmaniasis, and with asymptomatic infection after stimulation of whole blood with the soluble *Leishmania* and recombinant antigens. These results are represented as a heat map in Figure 3.

| **Analyte** | **Antigen** | **CVL** | **CCL** | **AS** | **HC** |
| --- | --- | --- | --- | --- | --- |
| **IFN-γ** | **SLA** | 100.0% (10/10) | 100.0/ (14/14) | 88.2% (15/17) | 0% (0/14) |
|  | **ENSC** | 70.0% (7/10) | 57.1% (8/14) | 40.0% (6/15) | 7.1% (1/14) |
|  | **NSC** | 50.0% (5/10) | 57.1% (8/14) | 53.3% (8/15) | 7.1% (1/14) |
|  | **ΔCpB** | 90.0 % (9/10) | 71.4% (10/14) | 53.3% (8/15) | 7.1% (1/14) |
| **TNF** | **SLA** | 80.0% (8/10) | 64.3% (9/14) | 73.3% (11/15) | 14.3% (2/14) |
|  | **ENSC** | 40.0% (4/10) | 64.3% (9/14) | 13.3% (2/15) | 7.1% (1/14) |
|  | **NSC** | 40.0% (4/10) | 57.2% (8/14) | 13.3% (2/15) | 0% (0/14) |
|  | **ΔCpB** | 60.0% (6/10) | 71.4% (10/14) | 13.3% (2/15) | 14.3% (2/14) |
| **IL-2** | **SLA** | 50.0% (5/10) | 85.7% (12/14) | 100% (15/15) | 7.1% (1/14) |
|  | **ENSC** | 10.0% (1/10) | 42.9% (6/14) | 60.0% (9/15) | 7.1% (1/14) |
|  | **NSC** | 20.0% (2/10) | 42.9% (6/14) | 60.0% (9/15) | 7.1% (1/14) |
|  | **ΔCpB** | 0.0% (0/10) | 42.9% (6/14) | 53.3% (8/15) | 7.1% (1/14) |
| **IP-10** | **SLA** | 90.0% (9/10) | 57.1% (8/14) | 100% (15/15) | 14.3% (2/14) |
|  | **ENSC** | 90.0% (9/10) | 50.0% (7/14) | 60.0% (9/15) | 14.3% (2/14) |
|  | **NSC** | 60.0% (6/10) | 42.9% (6/14) | 60.0% (9/15) | 21.4% (3/14) |
|  | **ΔCpB** | 40.0% (4/10) | 50.0% (7/14) | 53.3% (8/15) | 21.4% (3/14) |
| **IL-10** | **SLA** | 40% (4/10) | 64.3% (9/14) | 6.7% (1/15) | 7.1% (1/14) |
|  | **ENSC** | 10.0% (1/10) | 42.86% (6/14) | 6.7% (1/15) | 7.1% (1/14) |
|  | **NSC** | 30.0% (3/10) | 64.3% (9/14) | 6.7% (1/15) | 7.1% (1/14) |
|  | **ΔCpB** | 40.0% (4/10) | 57.14% (8/14) | 6.7% (1/15) | 7.1% (1/14) |
| **Granzyme B** | **SLA** | 80.0% (8/10) | 42.9 % (6/14) | 33.3% (5/15) | 7.1% (1/14) |
|  | **ENSC** | 0.0% (0/10) | 50.0% (7/14) | 26.7% (4/15) | 21.4% (3/14) |
|  | **NSC** | 10.0% (1/10) | 28.6% (4/14) | 0.0% (0/15) | 14.3% (2/14) |
|  | **ΔCpB** | 20% (2/10) | 21.4% (3/14) | 0.0% (0/15) | 14.3% (2/14) |

CVL= subjects cured of visceral leishmaniasis; CCL= subjects cured of cutaneous leishmaniasis; AS= subjects with asymptomatic *Leishmania* infection.

**References**

1. Aguilar-Be I, da Silva Zardo R, Paraguai de Souza E, Borja-Cabrera GP, Rosado-Vallado M, et al. Cross-protective efficacy of a prophylactic Leishmania donovani DNA vaccine against visceral and cutaneous murine leishmaniasis. Infect Immun. 2005;73(2):812–9.

2. Skeiky YA, Benson DR, Guderian JA, Whittle JA, Bacelar O, et al. Immune responses of leishmaniasis patients to heat shock proteins of Leishmania species and humans. Infect Immun. 1995;63(10):4105–14.

3. Coler RN, Duthie MS, Hofmeyer KA, Guderian J, Jayashankar L, et al. From mouse to man: safety, immunogenicity and efficacy of a candidate leishmaniasis vaccine LEISH-F3+GLA-SE. Clin Transl Immunology. 2015;4(4):e35.

4. Duthie MS, Pereira L, Favila M, Hofmeyer KA, Reed SJ, et al. A defined subunit vaccine that protects against vector-borne visceral leishmaniasis. NPJ Vaccines. 2017;2:23.

5. Chenik M, Louzir H, Ksontini H, Dilou A, Abdmouleh I, et al. Vaccination with the divergent portion of the protein histone H2B of Leishmania protects susceptible BALB/c mice against a virulent challenge with Leishmania major. Vaccine. 2006;24(14):2521–9.

6. Probst P, Stromberg E, Ghalib HW, Mozel M, Badaro R, et al. Identification and characterization of T cell-stimulating antigens from Leishmania by CD4 T cell expression cloning. J Immunol. 2001;166(1):498–505.
